# Gene expression signatures differentiating major depressive disorder from subsyndromal symptomatic depression

**DOI:** 10.18632/aging.202995

**Published:** 2021-05-08

**Authors:** Guoqin Hu, Shunying Yu, Chengmei Yuan, Wu Hong, Zuowei Wang, Ran Zhang, Dongxiang Wang, Zezhi Li, Zhenghui Yi, Yiru Fang

**Affiliations:** 1 Clinical Research Center, Shanghai Mental Health Center, Shanghai Jiao Tong University School of Medicine, Shanghai 200030, China; 2 Department of Psychiatry, Huangpu District Mental Health Center, Shanghai 200023, China; 3 Department of Psychiatry, Hongkou District Mental Health Center, Shanghai 200083, China; 4 Department of Neurology, Ren Ji Hospital, Shanghai Jiao Tong University School of Medicine, Shanghai 200127, China; 5 CAS Center for Excellence in Brain Science and Intelligence Technology, Shanghai 20000, China; 6 Shanghai Key Laboratory of Psychotic Disorders, Shanghai 201108, China

**Keywords:** major depressive disorder, subsyndromal symptomatic depression, co-expression analysis, specific analysis

## Abstract

Subsyndromal symptomatic depression (SSD) and major depressive disorder (MDD) have been classified as distinct diseases, due to their dissimilar gene expression profiles and responses to venlafaxine. To identify specific biomarkers of these two diseases, we conducted a secondary analysis of the gene expression signatures of SSD patients, MDD patients and healthy controls (n=8/group) from the study of Yi et al. Global, individual, specific, enrichment and co-expression analyses were used to compare the transcriptomic profiles of peripheral blood lymphocytes from the three groups. The global and individual analyses revealed that different genes were up- and downregulated in the SSD and MDD groups. Through our specific analysis, we identified 1719 and 3278 differentially expressed genes specifically associated with MDD and SSD, respectively. Enrichment and co-expression analyses demonstrated that the genes specific to MDD were enriched in pathways associated with hormone levels and immune responses, while those specific to SSD were associated with immune function. The specific hub gene for the MDD co-expression network was transmembrane protein 132B (TMEM132B), while the hub genes for SSD were actin-related protein 2/3 complex (ARPC2) and solute carrier family 5 member 5 (SLC5A5). This bioinformatic analysis has provided potential biomarkers that can distinguish SSD from MDD.

## INTRODUCTION

It has been estimated that 10% of people will suffer from depression at some time in their life [1]. Appropriate subtyping of depression can help to predict whether a patient will respond to antidepressants [2]. Major depressive disorder (MDD) is characterized by two core symptoms – loss of interest and loss of happiness – as well as other symptoms such as hopelessness, negative emotions, sleeping problems, anorexia and low energy levels, sustained for at least two weeks [3, 4]. About 15% of patients with MDD eventually die by suicide, although most patients can recover if they receive proper treatment [5].

Another type of depressive disorder, subsyndromal symptomatic depression (SSD), may occur before the onset of depression. Judd et al. [6] first proposed the concept of SSD, defining it as the presence of two or more depressive symptoms, lasting for at least two weeks and linked to damaged social function, but not accompanied by a depressed mood or anhedonia. The one-year prevalence rate of SSD is 8.4% [6]. SSD is an important indicator of disability and dysfunction, and the lifetime suicide attempt rate for SSD patients is 10.1%, comparable to that for MDD patients [7]. To date, there is no empirical information about the clinical course of SSD, but there are the following possibilities: SSD may be a self-limited disease that disappears over time; SSD may be a prodrome of MDD or dysthymia; SSD may be an incomplete recovery from MDD or dysthymia; or SSD may be a chronic, low-grade depressive mood pathological state [8–10]. Therefore, SSD is worthy of further research.

Previous genetic studies have suggested that SSD and MDD have overlapping genetic pathophysiologies, but the precise mechanisms of these two diseases have not been fully elucidated. Yi et al. [11] first used whole-genome mRNA microarray analyses of leukocytes to distinguish drug-free first-episode SSD patients from MDD patients and matched controls (n=8/group) based on their gene expression profiles. SSD and MDD patients had different genomic signatures, and a 48-gene model had the best performance in classifying SSD, MDD and healthy control subjects. Yang et al. [12] used quantitative real-time PCR to assess the mRNA levels of these 48 genes in peripheral blood samples from SSD, MDD and healthy control subjects (n=60/group), and found that three genes: domain-containing 84 (CD84*)*, striatin (STRN*)* and cystinosin (CTNS) were differentially expressed among the groups. In another study [2], differential co-expression and regulation analyses of peripheral blood lymphocytes suggested that six differentially regulated genes: fos-related antigen 1 (FOSL1*)*, serum response factor (SRF*)*, *JUN*, transcription factor activating enhancer binding protein 4 (TFAP4*)*, SRY-box transcription factor 9 (SOX9*)* and hepatic leukemia factor (HLF) and sixteen transcription factor-to-target differentially co-expressed gene links were the key differential factors in MDD, whereas one differentially regulated gene POZ/BTB and AT hook containing zinc finger 1 (PATZ1) and eight transcription factor-to-target differentially co-expressed gene links were the key differential factors in SSD. Overall, no target genes overlapped between MDD and SSD, and venlafaxine was found to significantly alter the gene expression profiles of MDD patients, but not SSD patients [2]. Using weighted gene co-expression network analyses, Geng et al. [13] identified 11 modules from 9427 differentially expressed genes (DEGs) in SSD. Gene Ontology (GO) and Kyoto Encyclopedia of Genes and Genomes (KEGG) pathway analyses demonstrated that the inflammatory response and type II diabetes mellitus were enriched in SSD, and 5'-nucleotidase domain containing 1 (NT5DC1*)*, Small G protein signaling modulator 2 (SGSM2*)* and MYC binding protein (MYCBP*)* were ultimately identified as significant hub genes. Hori et al. [14] measured gene expression in 14 medication-free moderate MDD subjects and 14 healthy controls, and found that 317 DEGs mapped to the ‘synaptic transmission’ pathway.

Objective and convenient biomarkers are needed to hasten the recognition of SSD, reduce its conversion to MDD and precisely treat different subtypes of depression. In this study, we determined the specific genes and enrichment pathways associated with MDD and SSD. Then, we constructed specific gene co-expression networks and identified highly specific hub genes of MDD and SSD.

## RESULTS

### Patient demographics

This study was a secondary analysis of data originally published by Yi et al. [11]. Drug-free first-episode MDD and SSD patients (n=8/group) and healthy controls (n=8) were enrolled. Both age and sex were matched among the groups (Table 1). The Structured Clinical Interview for the Diagnostic and Statistical Manual of Mental Disorders-Fourth Edition (SCID) and the 17-item Hamilton Rating Scale for Depression (HRSD-17) were administered to all subjects by two experienced psychiatrists with senior positions (interrater reliability, kappa = 0.87).

**Table 1:** General demographics of patients and healthy controls.

**Group**	**Age range (years)**
MDD	
1	25-30
2	25-30
3	25-30
4	35-40
5	25-30
6	30-35
7	30-35
8	40-45
SSD	
1	25-30
2	25-30
3	25-30
4	35-40
5	25-30
6	25-30
7	35-40
8	40-45
Healthy control	
1	20-25
2	25-30
3	25-30
4	35-40
5	25-30
6	30-35
7	30-35
8	40-45

### Global analysis of mRNAs

Peripheral blood samples from the three groups of patients were subjected to microarray analyses so that mRNA levels could be compared among the groups. The most striking differences in mRNA levels were observed between the SSD and healthy control groups. Among 54,675 mRNAs, 9427 were differentially expressed between the SSD and healthy control groups (p ≤ 0.05), with a tendency to be upregulated in the SSD group (6.76% increase). Of the genes with larger expression changes (fold-change > 1.30 or < 0.77), 3789 were upregulated and 3170 were downregulated in the SSD group compared with the healthy control group.

Next, we compared MDD patients with healthy controls, we observed relatively few global mRNA changes. Of the 54,675 mRNAs, 4125 were differentially expressed between the MDD and healthy control groups (p ≤ 0.05), with a tendency to be upregulated in the MDD group (0.02% increase). Among the genes with larger expression changes (fold-change > 1.3 or < 0.769), 1434 were upregulated and 1677 were downregulated in the MDD group compared with the healthy control group.

When we compared global mRNA levels between MDD and SSD patients. We found that 9262 of the 54,675 mRNAs were differentially expressed between these two groups (p ≤ 0.05), with a tendency to be upregulated in the MDD group (7.08% increase). Among the genes with larger expression changes (fold-change > 1.30 or < 0.77), 3100 were upregulated and 3900 were downregulated in the MDD group compared with the SSD group.

The overall dataset reflected obvious, widespread mRNA changes in SSD patients vs MDD patients. Thus, altered mRNA expression may be regarded as a biomarker of SSD that is adaptive and homeostatic but lacking in MDD patients.

### Analysis of individual mRNAs

Of the 9427 mRNAs that were significantly differentially expressed between SSD patients and controls, the top ten upregulated and downregulated genes are exhibited in Table 2. FRAS1-related extracellular matrix 3 (FREM3) on chromosome 11: 62040583-62047156 was the most strongly upregulated gene (fold-change: 14.74; p-value: 0.03), while C-C motif chemokine ligand 20 (CCL20*)* on chromosome 2: 228386813-228390494 was the most strongly downregulated gene (fold-change: 0.05; p-value: 0.02).

**Table 2:** Significantly up- and downregulated mRNAs in SSD patients vs. healthy controls.

	**Gene**	**Fold-change**	**p-value**	**chromosome**
	Upregulated			
1	*FREM3*	14.73884566	0.033489922	chr11: 62040583-62047156
2	*PDZRN3*	9.36926631	0.014126602	chr3: 73759272-73759736
3	*GALP*	9.059542668	0.003601305	chr19: 61379200-61388956
4	*POU4F3*	8.568801129	0.021930619	chr5: 145698868-145700200
5	*ESPN*	7.6434056	0.007291285	chr1: 6407587-6442993
6	*FN1*	7.528425974	0.037148336	chr2: 215933832-216008690
7	*BTBD8*	7.463212882	0.023074933	chr1: 92318480-92385933
8	*FARP2*	7.203860206	0.008283210	chr2: 241944383-242054117
9	*NTRK3*	7.027205922	0.000779235	chr15: 86207772-86209836
10	*IGF1R*	6.921653028	0.025073337	chr15: 99276454-99276946
	Downregulated			
1	*CCL20*	0.050188723	0.020152367	chr2: 228386813-228390494
2	*AHNAK*	0.09476463	0.009823222	chr11: 62039949-62050658
3	*LOC399744*	0.117500665	0.000168497	chr10: 38732122-38777664
4	*PFAAP5*	0.119307294	0.014539339	chr13: 31982654-31985621
5	*CCDC102B*	0.12814995	0.025351322	chr18: 65030493-65032564
6	*SORBS2*	0.128267303	0.000597935	chr4: 186798113-186799907
7	*LNPEP*	0.14701882	0.004190173	chr5: 96394114-96394977
8	*RAB3C*	0.157130794	0.014039095	chr5: 58188365-58188910
9	*AJ276555*	0.157265173	0.011187684	chr12: 109859370-109859638
10	*CPAP*	0.163601794	0.010409793	chr13: 24361345-24376132

The top ten upregulated and downregulated genes among the 4125 significantly differentially expressed mRNAs between MDD patients and controls are shown in Table 3. Olfactory receptor 2H1 (OR2H1) on chromosome 6: 29537525-29538476 was the most strongly upregulated gene (fold-change: 10.50; p-value: 0.04), while *CCL20* on chromosome 2: 228386813-228390494 was the most strongly downregulated gene (fold-change: 0.09; p-value: 0.02).

**Table 3:** Significantly up- and downregulated mRNAs in MDD patients vs. healthy controls.

	**Gene**	**Fold-change**		**p-value**	**chromosome**
	Upregulated				
1	*OR2H1*	10.49930199		0.036466554	chr6: 29537525-29538476
2	*PDZRN3*	8.040524786		0.035864269	chr3: 73759272-73759736
3	*NRXN1*	7.266546337		0.027104021	chr2: 49999506-50001065
4	*AQP4-AS1*	6.583444561		0.001625302	chr18: 26737858-26738017
5	*ADAM30*	6.323491911		0.033936130	chr1: 120237954-120238204
6	*TAX1BP3*	6.228224117		0.009671162	chr17: 3517422-3518603
7	*POU4F3*	6.121667165		0.042860783	chr5: 145698868-145700200
8	*ESPN*	6.02225045		0.011621354	chr1: 6407587-6442993
9	*NTRK3*	5.924995635		0.002248940	chr15: 86207772-86209836
10	*CPE*	5.858833617		0.009855088	chr4: 166519543-166638926
	Downregulated				
1	*CCL20*	0.093343424		0.018734019	chr2: 228386813-228390494
2	*HLA-DQB1*	0.109670469		0.021685769	chr6: 32735224-32742572
3	*RP1*	0.115663331		0.009439945	chr6: 163733604-163738866
4	*SYT1*	0.132274079		0.007769112	chr12: 78317217-78317562
5	*CHES1*	0.155760903		0.029942786	chr14: 87671581-87672158
6	*RAB32*	0.160788193		0.028362683	chr6: 146912587-146915288
7	*RAB7A*	0.164235711		0.024497067	chr3: 130013902-130015623
8	*LOC101927760*	0.164256336		0.028223721	chr10: 119991065-119991576
9	*CXCL3*	0.16776403		0.011589400	chr4: 75121177-75123269
10	*H2AFY2*	0.168078772		0.017307962	chr10: 71515566-71516055

Table 4 displays the top 10 upregulated and downregulated genes among the 9262 differentially expressed mRNAs between MDD and SSD patients. *AHNAK* on chromosome 11: 62039949-62050658 was the most strongly upregulated gene (fold-change: 12.01; p-value: 0.0094), whereas galanin-like peptide (*GALP)* on chromosome 19: 61379200-61388956 was the most strongly downregulated gene (fold-change: 0.097; p-value: 0.0022).

**Table 4:** Significantly up- and downregulated mRNAs in MDD patients vs. SSD patients.

	**Gene**	**Fold-change**	**p-value**	**chromosome**
	Upregulated			
1	AHNAK	12.00684864	0.009447029	chr11: 62039949-62050658
2	EDNRB	8.171230689	0.001870225	chr13: 77370240-77390755
3	LOC284701	7.057598609	0.010444377	chr1: 226221713-226228812
4	BC032034	6.557337529	0.001381698	chr3: 180301847-180348451
5	LNPEP	6.21575868	0.011049362	chr5: 96394114-96394977
6	IGFBP5	6.037622489	0.025706624	chr2: 217248219-217249383
7	PFAAP5	5.982076617	0.040062454	chr13: 31982654-31985621
8	LOC102724612	5.894702597	0.042944315	chr8: 64540959-64547620
9	PDGFRA	5.740653281	0.002692000	chr4: 54837254-54843018
10	LOC728852	5.737907484	0.022468675	chr14: 30959714-30991839
	Downregulated			
1	GALP	0.09672831	0.002222873	chr19: 61379200-61388956
2	RANBP2L1	0.107935435	0.009097415	chr2: 111085983-111086739
3	FIP1L1	0.115811265	0.001991012	chr11: 37991635-37992296
4	SLC9A7	0.132974447	0.002015236	chrX: 46349700-46503303
5	GUCA1C	0.134880497	0.003687287	chr3: 110109339-110155310
6	RH17876	0.138002894	0.014801866	chr1: 34743617-34744982
7	USP26	0.150844724	0.016120346	chrX: 131987172-131989966
8	SERPINB4	0.153862372	0.010070519	chr18: 59455931-59461796
9	CARD8	0.153939137	0.013866022	chr19: 53398216-53414013
10	ARHGAP29	0.154003298	0.005056514	chr1: 94414770-94434085

### Specific analysis

**Figure 1:**
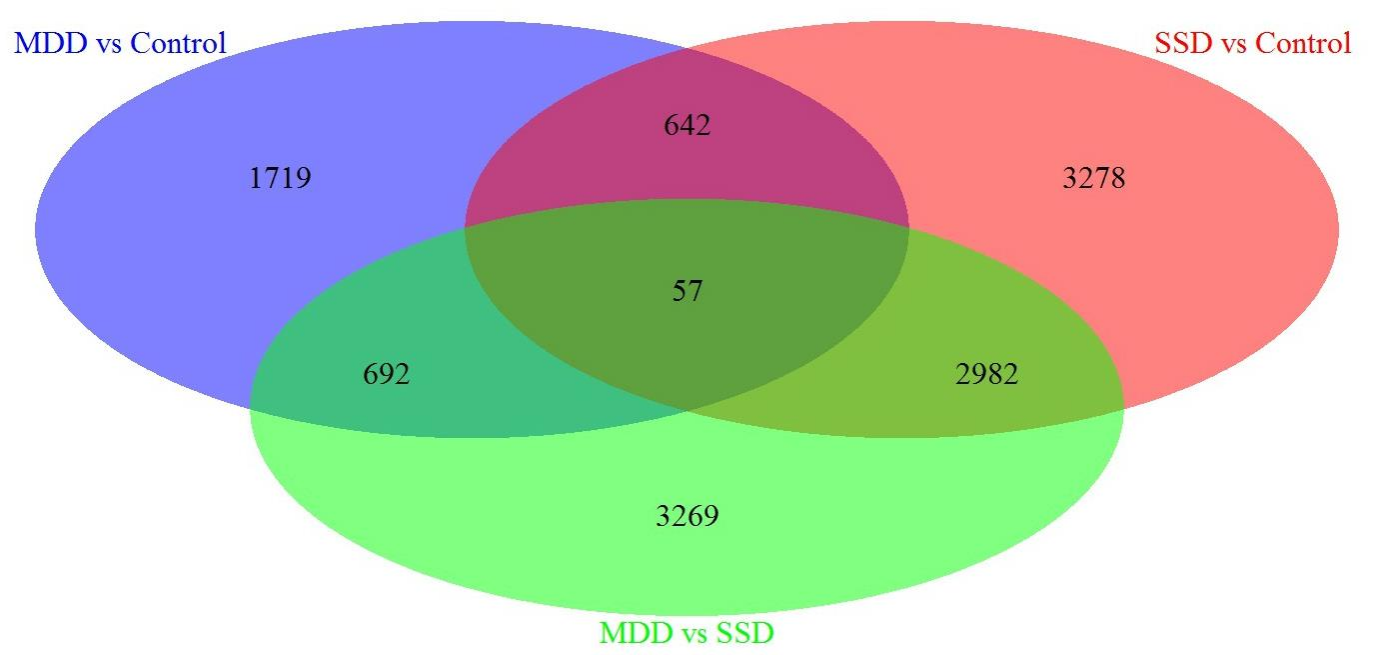
**Venn diagram of DEGs in blood samples from MDD, SSD and healthy control subjects.** Three-way Venn diagram of the total number of significantly DEGs (p ≤ 0.05) in the MDD vs. healthy control (HC), SSD vs. HC and MDD vs. SSD comparisons. The numbers of genes that are unique for each disease are shown in the circle beside the Venn diagram. The numbers of genes shared are indicated at the intersections of the circles in the Venn diagram.

Next, we performed a specific analysis, which identified 3278 DEGs specifically associated with SSD patients vs. controls, 1719 DEGs specifically associated with MDD patients vs. Controls and 3269 DEGs specifically associated with MDD patients vs. SSD patients (Refer to Venn diagram for details, Figure 1).

### Functional enrichment analysis of genes from the specific analysis

**Figure 2:**
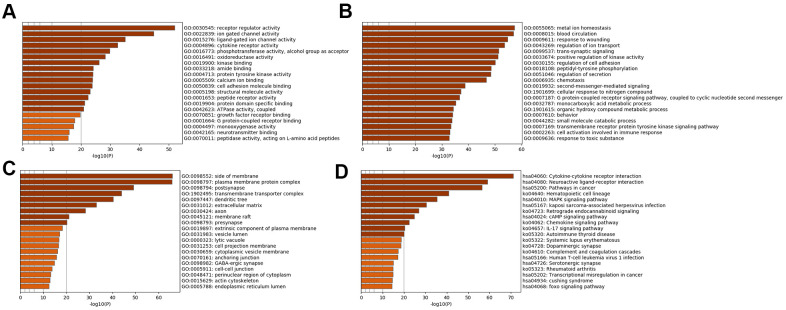
**Bar graph of enriched terms across input SSD-specific genes, colored by p-values.** Provided gene identifiers were first converted into corresponding H. sapiens Entrez gene IDs using the latest version of the database (last updated on 2020-09-16). If multiple identifiers corresponded to the same Entrez gene ID, they were considered as a single Entrez gene ID in downstream analyses. For each given gene list, pathway and process enrichment analyses were performed using the following ontology sources: (**A**) GO MFs; (**B**) GO BPs; (**C**) GO CCs; (**D**) KEGG pathways. All genes in the genome were used as the enrichment background. “Log10(P)” is the p-value in log base 10.

**Figure 3:**
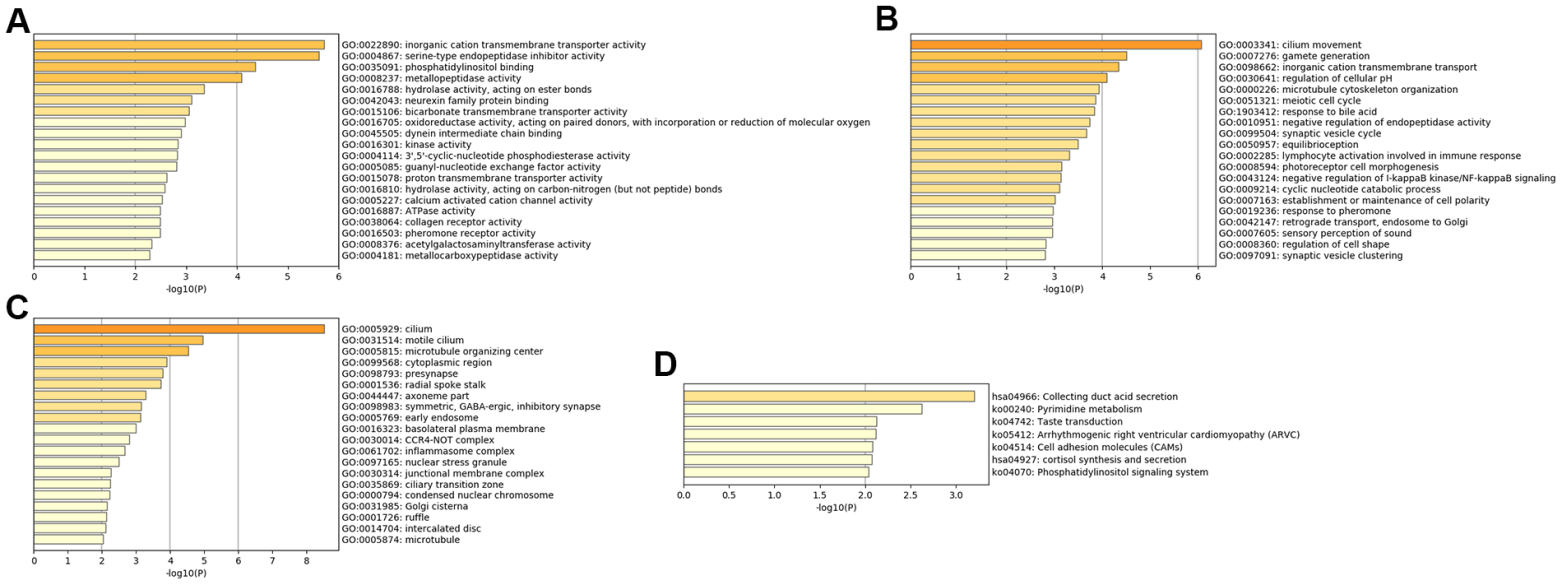
**Bar graph of enriched terms across input MDD-specific genes, colored by p-values.** Provided gene identifiers were first converted into corresponding H. sapiens Entrez gene IDs using the latest version of the database (last updated on 2020-09-16). If multiple identifiers corresponded to the same Entrez gene ID, they were considered as a single Entrez gene ID in downstream analyses. For each given gene list, pathway and process enrichment analyses were performed using the following ontology sources: (**A**) GO MFs; (**B**) GO BPs; (**C**) GO CCs; (**D**) KEGG pathways. All genes in the genome were used as the enrichment background. “Log10(P)” is the p-value in log base 10.

Subsequently, enrichment analyses were performed to determine the GO molecular functions (MFs), GO biological processes (BPs), GO cellular components (CCs) and KEGG pathways of the genes in the specific analysis. Figure 2 displays the pathways of SSD, while Figure 3 displays the pathways of MDD. The MFs of SSD were mainly enriched in ‘neurotransmitter binding’, ‘G protein-coupled receptor binding’, ‘growth factor receptor binding’, ‘ATPase activity, coupled, calcium ion binding’, ‘protein tyrosine kinase activity’, ‘cytokine receptor activity’, ‘ligand-gated ion channel activity’ and ‘ion gated channel activity’ (Figure 2A). The BPs of SSD were mainly enriched in ‘cell activation involved in immune response’, ‘transmembrane receptor protein tyrosine kinase signaling pathway’, ‘G protein-coupled receptor signaling pathway’, ‘coupled to cyclic nucleotide second messenger’, ‘second-messenger-mediated signaling’, ‘regulation of secretion’, ‘peptidyl-tyrosine phosphorylation’, ‘positive regulation of kinase activity’ and ‘trans-synaptic signaling’ (Figure 2B). The CCs of SSD were mainly enriched in ‘endoplasmic reticulum lumen’, ‘actin cytoskeleton’, ‘perinuclear region of cytoplasm’, ‘cell-cell junction’ and ‘GABA-ergic synapse’ (Figure 2C). The KEGG pathways of SSD were mainly enriched in ‘Cushing syndrome’, ‘transcriptional misregulation in cancer’, ‘rheumatoid arthritis’, ‘serotonergic synapse’, ‘human T-cell leukemia virus 1 infection’, ‘complement and coagulation cascades’, ‘dopaminergic synapse’, ‘systemic lupus erythematosus’, ‘autoimmune thyroid disease’, ‘IL-17 signaling pathway’, ‘chemokine signaling pathway’, ‘cAMP signaling pathway’, ‘retrograde endocannabinoid signaling’, ‘Kaposi sarcoma-associated herpesvirus infection’ and ‘MAPK signaling pathway’ (Figure 2D).

The MFs of MDD were mainly enriched in ‘ATPase activity’, ‘calcium activated cation channel activity’, ‘3',5'-cyclic-nucleotide phosphodiesterase activity’, ‘kinase activity’, ‘hydrolase activity’, ‘acting on ester bonds’ and ‘inorganic cation transmembrane transporter activity’ (Figure 3A). The BPs of MDD were mainly enriched in ‘response to pheromone’, ‘lymphocyte activation involved in immune response’ and ‘inorganic cation transmembrane transport’ (Figure 3B). The CCs of MDD were mainly enriched in the ‘CCR4-NOT complex’ and ‘symmetric, GABA-ergic, inhibitory synapse’ (Figure 3C). The KEGG pathways of MDD were mainly enriched in ‘cortisol synthesis and secretion’, ‘cell adhesion molecules’, ‘arrhythmogenic right ventricular cardiomyopathy’ and ‘pyrimidine metabolism’ (Figure 3D).

### Co-expression analysis of genes from the specific analysis

**Figure 4:**
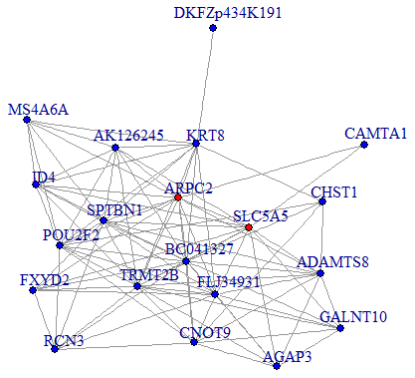
**Co-expression subnetworks of SSD-associated genes.** Nodes in the network represent genes, and edges represent significant co-expression (≥ 0.80) between two genes. Different colors indicate different strengths of co-expression. Genes colored in red are hub genes, and genes colored in blue are corresponding genes.

**Figure 5:**
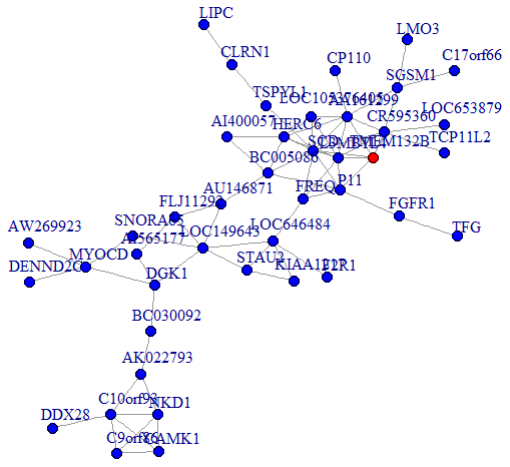
**Co-expression subnetworks of MDD-associated genes.** Nodes in the network represent genes, and edges represent significant co-expression (≥ 0.80) between two genes. Different colors indicate different strengths of co-expression. The gene colored in red is the hub gene, and genes colored in blue are corresponding genes.

We then performed a co-expression analysis, which suggested that there was a significantly higher correlation and connectivity of gene expression in SSD patients than in MDD patients or healthy controls. The co-expression analysis also indicated that SSD and MDD patients had different transcription signatures. Thus, we constructed normal gene co-expression networks using all the specifically differentially expressed mRNAs of the MDD and SSD patients. The most concentrated differentially expressed mRNAs of the SSD patients constituted a network of 20 nodes and 72 connections (Figure 4), while those of the MDD patients constituted a network of 42 nodes and 66 connections (Figure 5). The hub genes for the SSD co-expression network were actin-related protein 2/3 complex (ARPC2) and solute carrier family 5 member 5 (SLC5A5), located at chr2: 218790364-218827076 and chr19: 17843906-17865777, respectively. The hub gene for the MDD co-expression network was transmembrane protein 132B (TMEM132B)*,* located at chr12: 124709076-124709542. The network graphs revealed that the nodes and connections in the SSD subnetwork were significantly richer than those in the MDD subnetwork.

Overall, different gene expression characteristics were found in peripheral blood samples from SSD and MDD patients in this study. The hub genes detected in this study may be associated with the etiology, diagnosis and treatment of MDD and SSD.

## DISCUSSION

In the original study on which this study was based, Yi et al. [11] identified 1456 DEGs between SSD patients and normal controls, along with 149 DEGs between MDD patients and normal controls, both at a significance level of p < 0.01. By applying stricter thresholds to their intergroup comparisons, the authors identified a signature of 63 differentially expressed genes between SSD patients and controls (adjusted p = 1.0E-4), a signature of 30 differentially expressed genes between MDD patients and controls (adjusted p = 5.0E-4), and a signature of 123 differentially expressed genes between SSD and MDD patients (adjusted p = 1.0E-4). In our re-analysis of the data, we selected a significance threshold that would include more genes, in order to gain a more comprehensive understanding of the biological markers of MDD and SSD. At a significance level of p ≤ 0.05, 9427 genes were differentially expressed between SSD patients and healthy controls, 4125 were differentially expressed between MDD patients and healthy controls and 9262 were differentially expressed between MDD and SSD patients.

When we individually analyzed the differentially expressed mRNAs between the groups, we found that *FREM3* was the most strongly upregulated and *CCL20* was the most strongly downregulated gene in SSD patients vs. healthy controls. These two genes have previously been linked to mental diseases. *FREM3* was significantly associated with MDD in a genome-wide association study [15], and Nikolova et al. reported that depression risk factors such as slower perceptual processing speeds and reduced reactivity to environmental stimuli may be due to reduced FREM3 expression [16]. The C-C motif chemokine 20 protein encoded by *CCL20* is an important Th17 mediator that is involved in inflammatory bowel disease [17], indicating that *CCL20* may be associated with immune function.

For MDD patients vs. healthy controls, *OR2H1* was the most strongly upregulated and *CCL20* was the most strongly downregulated gene. In a parallel case-control study, Orozco et al. found three new independent loci: Zinc finger 391(ZNF391*)*, *OR2H1* and *c6orf26-rdbp* in the major histocompatibility complex region that were associated with rheumatoid arthritis [18]. Patients with rheumatoid arthritis were found to have a greater risk of depression than normal controls and patients with remitted vasculitis, and *CCL20* (Th17) was reported to be significantly upregulated in active vasculitis patients [19]. Some studies have demonstrated that olfaction disorders and immune diseases are associated with depression, possibly because all these diseases involve excess production of inflammatory cytokines and eicosanoids [20].

In our comparison between MDD vs. SSD patients, *AHNAK* was the most strongly upregulated and *GALP* was the most strongly downregulated gene. Constitutive *AHNAK* knockout mice and forebrain glutamatergic neuron-selective *AHNAK* knockout mice were found to have a depression-like behavioral phenotype, whereas parvalbumin interneuron-selective *AHNAK* knockout mice displayed an antidepressant-like behavioral phenotype [21]. Thus, *AHNAK* seems to control depressive behavior. *GALP* is generated by neurons in the median eminence and basomedial arcuate nucleus [22], and is associated with immune function [23]. The differential expression of these genes between MDD and SSD patients indicates that these diseases are associated with the immune system. To the best of our knowledge, this is the first study to analyze individual candidate genes associated with MDD and SSD.

We also identified new candidate genes that were only enriched in MDD or SSD patients. In our Venn diagram analysis, 1719 DEGs, 3278 DEGs and 3269 DEGs were specifically associated with comparisons of MDD patients vs. controls, SSD patients vs. controls and MDD patients vs. SSD patients, respectively. Depression and lack of interest are core symptoms of MDD, but not of SSD; thus, the DEGs specifically associated with MDD may be involved in the underlying pathological mechanisms of depression or anhedonia.

Our functional enrichment analysis demonstrated that altered hormone levels may increase the risk of MDD, while inflammatory responses may contribute to SSD. In MDD patients, GO analysis demonstrated that ‘response to pheromone’ was the most enriched BP, and KEGG pathway analysis indicated that altered ‘pyrimidine metabolism’ may increase the risk of MDD. Early studies on the projection of melanin-concentrating hormone (MCH)-ergic neurons and the distribution of melanin-concentrating hormone receptor 1 (MCH-R1) suggested that MCH may regulate emotions [24, 25]. MCH directly contributes to depression-like behaviors by inhibiting the monoaminergic neurotransmitter function of the dorsal raphe nucleus and the locus coeruleus nucleus [26], and indirectly contributes to depression-like behaviors by regulating the sleep-wake cycle [27]. Also the hypothalamic-pituitary-thyroid [28] and hypothalamic-pituitary-adrenal axes [29] are altered in major depression. Uridine, a pyrimidine metabolite, has been shown to have antidepressant-like activities in mice [30].

In SSD patients, GO analysis indicated that ‘cell activation involved in immune response’ was the most enriched BP. KEGG pathway analysis demonstrated that inflammatory cytokines may increase the risk of SSD, as evidenced by the enrichment of ‘rheumatoid arthritis’, ‘serotonergic synapse’, ‘human T-cell leukemia virus 1 infection’, ‘complement and coagulation cascades’, ‘dopaminergic synapse’, ‘systemic lupus erythematosus’, ‘autoimmune thyroid disease’, ‘IL-17 signaling pathway’, ‘chemokine signaling pathway’ and ‘Kaposi sarcoma-associated herpesvirus infection’. The limited research thus far suggests that the immune system may contribute to the development of SSD. Geng et al. [13] found that GO terms such as ‘regulation of leukocyte migration’, ‘T cell-mediated immunity’ and ‘regulation of autophagy’ were enriched in SSD patients, along with KEGG pathways such as ‘Th17 cell differentiation’ and ‘the NOD-like receptor signaling pathway’.

Alterations in the peripheral immune system and subsequent overactivation of pro-inflammatory cytokines have long been associated with mood disorders [31, 32], leading to the proposal of a macrophage theory of depression [33]. In addition, continuous activation of the peripheral immune system due to cancer, systemic infections or autoimmune diseases may promote the development of major depression in vulnerable individuals [34]. Altered leukocyte function/number and elevated cytokine expression have been proposed as potential biomarkers of depression [35, 36] and post-traumatic stress disorder [37]. Moreover, anti-inflammatory drugs may have antidepressant effects in MDD patients [38]. ‘Cell activation involved in immune response’ was identified in our pathway analyses of both MDD and SSD patients. Although the relationship between SSD and MDD is unclear, previous studies have indicated that SSD is a subtype of depression and a transitory phenomenon in the depression spectrum with a high likelihood of transition to MDD [39–41]. Thus, genes involved in the ‘cell activation involved in immune response’ pathway may contribute to the pathogenesis of both MDD and SSD.

Our co-expression network analysis identified *ARPC2* and *SLC5A5* as hub genes contributing to SSD. Previously, *ARPC2* expression was found to be significantly elevated in gastric cancer tissues [42]. *SLC5A5* was reported to be downregulated in papillary and follicular thyroid cancer [43, 44] and dysregulated in patients with neurotransmitter, endocrine and immune abnormalities [45]. These genes have also been associated with neuroplasticity, cognitive function and neuropsychiatric disease development [46]. The present study was the first to reveal the involvement of *ARPC2* and *SLC5A5* in SSD.

In the study of Yi et al. [11], peripheral blood leukocytes from MDD and SSD patients had different genomic signatures, and a 48-gene model was proposed to classify SSD patients, MDD patients and healthy controls. In the current study, we found some differences between the hub genes of SSD and MDD. Our co-expression network analysis suggested that *TMEM132B* participates in the pathogenesis of MDD as a hub gene. Peripheral blood *TMEM132B* mRNA expression was previously found to differ significantly between aneurysm patients and controls [47]. Aneurysms are known to be caused by immune illnesses, infections, acute or blunt injuries and atherosclerosis; thus, the alteration of *TMEM132B* in both MDD and aneurysm patients suggests that MDD is also linked to immunity and inflammation.

This study had several limitations. We used peripheral blood samples rather than brain tissues or cerebrospinal fluid to compare the expression profiles of MDD and SSD patients. Previous studies have shown that gene expression overlaps considerably between peripheral blood and the brain [48]. We chose to identify biomarkers from peripheral blood to circumvent several limitations of postmortem brain tissue and cerebrospinal fluid, such as invasive acquisition and low patient acceptance. Additional SSD and MDD samples will be needed to replicate our results. Quantitative real-time PCR should be used to verify the levels of the three differentially expressed hub genes in SSD and MDD patients, and cell or animal studies should be conducted to further explore the pathophysiological functions of these genes in SSD and MDD.

## MATERIALS AND METHODS

### Subjects

All subjects were recruited from the ward and clinic of Shanghai Mental Health Center, China. Our project was approved by the Institutional Review Board of Shanghai Mental Health Center in accordance with the World Medical Association’s Declaration of Helsinki. Informed consent was obtained from each subject before the study.

Candidates for the MDD group were required to meet the Diagnostic and Statistical Manual of Mental Disorders-Fourth Edition criteria for MDD and have scores ≥ 17 on the HRSD-17. The exclusion criteria were pregnancy and other special physical conditions. For inclusion in the SSD group, patients were required to have two or more depressive symptoms, exhibit social dysfunction, be free of anhedonia and a depressed mood, and have a total HRSD-17 score of 8-16 for approximately two weeks. Healthy control subjects were required to score ≤ 7 on the HRSD-17 and to have no severe physical illness. Additional details can be found in the original paper [11].

### Microarray analysis

Blood samples for mRNA and protein analyses were obtained after overnight fasting. Venous blood (5 mL) was collected between 7 and 9 a.m. in anticoagulant-free tubes. Blood for leukomonocyte extraction was collected from the whole blood using Ficoll-Paque PLUS reagent (GE Healthcare, IL, USA), and was transferred into fresh RNase/DNase-free microcentrifuge tubes with TRIzol (Invitrogen, CA, USA) before being stored at -80° C. Subsequently, total RNA was extracted using TRIzol [11]. The cDNA synthesis, cDNA hybridization, signal scanning, data acquisition and preliminary analysis were performed using the platform of the Affymetrix U133 Plus2.0 GeneChip oligonucleotide array. The raw expression data were standardized through robust multi-array averaging using Gene Spring Software 11.0 (Agilent Technologies, Santa CA, USA), and log2 transformation was applied. The details of the analysis have been published previously [11].

All data have been shared in the Gene Expression Omnibus (http://www.ncbi.nlm.nih.gov/geo/query/acc.cgi?acc=gse32280). We reused the GSE32280 dataset [11].

### Specific analysis

Specific mRNAs that were differentially expressed between the different pairs of groups were identified through a Venn diagram analysis using the “draw.triple.venn” package in R.

### Functional enrichment analysis of genes from the specific analysis

The CCs, MFs, BPs and KEGG pathways of the MDD- and SSD-specific genes were determined through GO and KEGG pathway enrichment analyses using Metascape (https://metascape.org/gp/index.html) [49]. Terms with p-values < 0.01, a minimum count of three and an enrichment factor > 1.5 (the ratio between the observed counts and the counts expected by chance) were collected and grouped into clusters based on their membership similarities. P-values were calculated based on the accumulative hypergeometric distribution [50]. Sub-trees with similarity values > 0.3 were considered to be a cluster. The most statistically significant term within a cluster was chosen to represent the cluster.

### Co-expression network from the specific analysis

The expression correlations of co-expressed genes were used to determine their total connectivity and identify gene-gene interactions in SSD and MDD. Highly correlated genes may be functionally related or involved in similar biological processes. We used the “igraph” package in the R platform to construct the co-expression network.
